# Supplementary figures and images for: Small-scale screening of anticancer drugs acting specifically on neural stem/progenitor cells derived from human-induced pluripotent stem cells using a time-course cytotoxicity test
Source: PeerJ. 2018 Jan 4;6:e4187. doi: 10.7717/peerj.4187 (PMC5756610; doi:10.7717/peerj.4187)

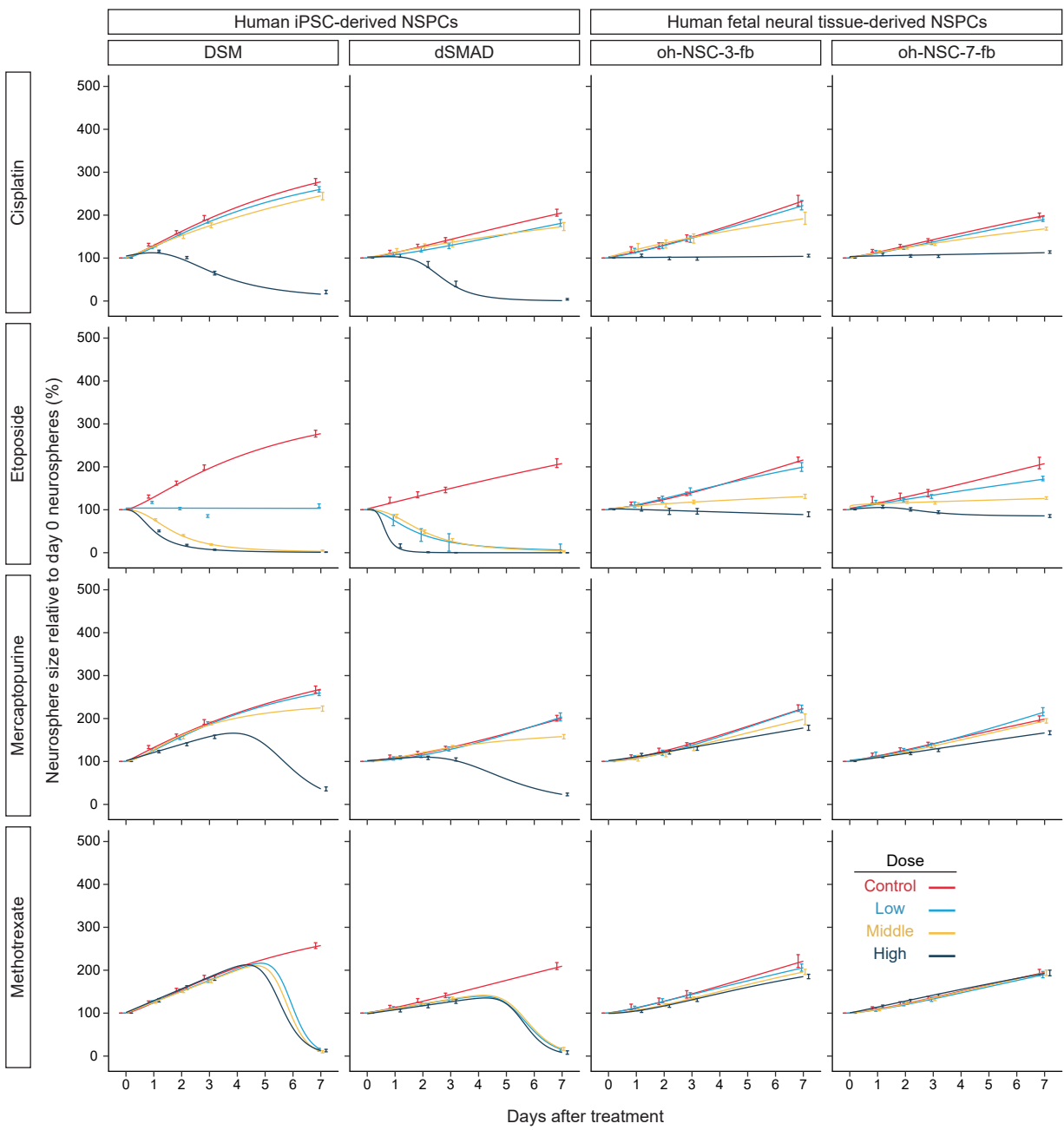

Supplement: Figure S1 — Colors indicate drug concentrations. The Brain–Cousens five-parameter model (BC.5) was used. Error bars represent the 95% CI. [file peerj-06-4187-s001.pdf]
