# Supplementary material for: Small-scale screening of anticancer drugs acting specifically on neural stem/progenitor cells derived from human-induced pluripotent stem cells using a time-course cytotoxicity test
Source: PeerJ. 2018 Jan 4;6:e4187. doi: 10.7717/peerj.4187 (PMC5756610; doi:10.7717/peerj.4187)
Supplement: Table S1 [file peerj-06-4187-s002.docx]

Table S1. The number of neurospheres used in the time-course cytotoxicity test

| Drug | | hiPSC-NSPCs | | hN-NSPCs | |
| --- | --- | --- | --- | --- | --- |
| Name | Dose | DSM | dSMAD | oh-NSC-3-fb | oh-NSC-7-fb |
| Cisplatin | Control | 140 | 75 | 76 | 100 |
|  | Low | 163 | 45 | 75 | 85 |
|  | Middle | 93 | 44 | 50 | 112 |
|  | High | 155 | 68 | 85 | 82 |
| Etoposide | Control | 114 | 69 | 117 | 91 |
|  | Low | 125 | 8 | 25 | 110 |
|  | Middle | 163 | 70 | 64 | 135 |
|  | High | 151 | 68 | 29 | 77 |
| Mercaptopurine | Control | 144 | 101 | 76 | 88 |
|  | Low | 161 | 81 | 35 | 88 |
|  | Middle | 162 | 122 | 35 | 106 |
|  | High | 151 | 116 | 94 | 100 |
| Methotrexate | Control | 119 | 114 | 73 | 43 |
|  | Low | 160 | 122 | 86 | 59 |
|  | Middle | 151 | 127 | 84 | 88 |
|  | High | 162 | 71 | 96 | 96 |
